# Successful treatment of gastropulmonary fistula in a patient with gastric diverticulum: A case report

**DOI:** 10.1097/MD.0000000000042152

**Published:** 2025-04-18

**Authors:** Tae-Hong Yoon, Han Sol Lee, Chul Ho Lee, Yun-Ho Jeon

**Affiliations:** a Department of Thoracic and Cardiovascular Surgery, Daegu Catholic University School of Medicine, Daegu, Republic of Korea.

**Keywords:** gastric diverticulum, gastropulmonary fistula

## Abstract

**Rationale:**

Gastropulmonary fistula (GPF) is defined as a communication pathway between the stomach and the lung. GPF is a rare condition, and there is no consensus regarding its treatment. GPF has been reported in only a small number of cases.

**Patient concerns:**

A 61-year-old male patient visited the emergency room due to abdominal pain and concurrent hematemesis that started 2 days prior. The patient reported a history of frequent vomiting attributed to chronic alcoholism and gastric ulcer-related bleeding. Multiple gastric ulceration and gastric diverticulum were diagnosed at that time.

**Diagnoses:**

GPF was diagnosed by a noncontrast-enhanced chest computed tomography scan after swallowing an oral contrast agent.

**Interventions:**

GPF was treated with surgical intervention. A left lower lobectomy was performed through a posterolateral thoracotomy at the left 6th intercostal space. The 2.5 cm diaphragmatic opening was closed using a pledgeted 2-0 Prolene suture. In the abdomen, a partial gastrectomy was performed via an upper median laparotomy.

**Outcomes:**

Successful surgical management of a PGF. Postoperative pneumonia developed on postoperative day (POD) 3, prompting a change in antibiotics. On POD20, an abdominal wound infection occurred, for which a vacuum-assisted closure system was applied. The patient began oral intake on POD33 and was discharged on POD56 after the removal of the tracheostomy tube.

**Lessons:**

This case demonstrates that surgical intervention should be considered the primary treatment option when managing a patient diagnosed with GPF.

## 1. Introduction

Gastropulmonary fistula (GPF) is defined as a communication pathway between the stomach and the lung. Anatomically, this condition typically occurs in the left lung, where the stomach comes into contact with the diaphragm. GPF is characterized by symptoms such as chest pain, shortness of breath, exacerbation of purulent coughing during oral intake, and left lower lobe (LLL) pneumonia. Further, these symptoms can be accompanied by hemoptysis.

The mechanism of GPF is direct gastric perforation due to trauma or transdiaphragmatic erosion to the basal lobe of the left lung from the stomach that is caused by inflammation. GPF is a rare complication, arising as a secondary consequence of leakage complications attributed to bariatric surgeries, such as sleeve gastrectomy. The leaked gastric contents from the subphrenic area can cause inflammation, which then triggers the development of GPF. Leaks occur after bariatric surgery in approximately 2% to 5% of cases, with the incidence of GPF at approximately 0.2%.^[1,2]^

In this case, we discussed the successful surgical management of GPF caused by a gastric diverticulum. The patient provided informed consent for the publication of this case.

## 2. Case report

**Figure 1.**
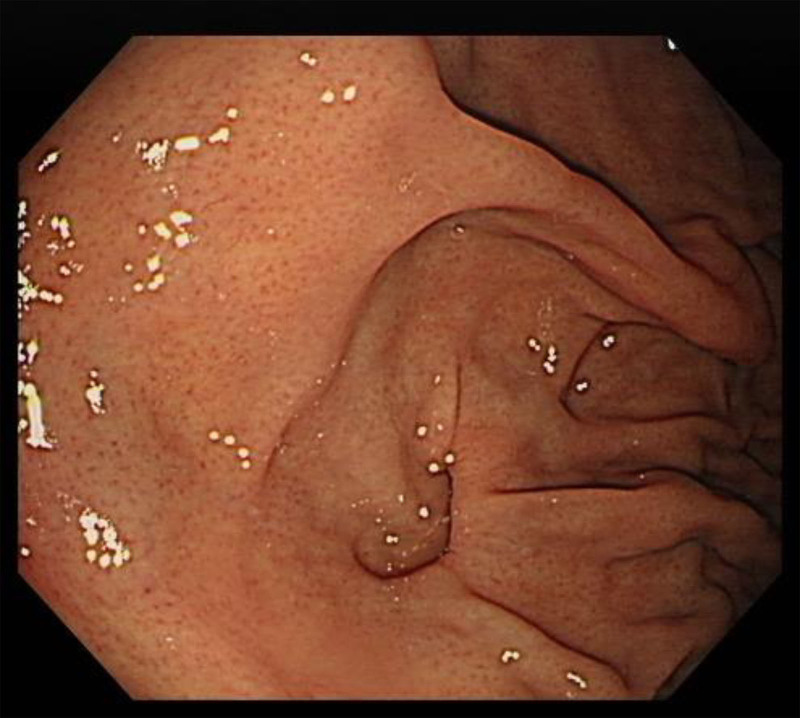
Gastric diverticulum at the fundus on esophagogastroduodenoscopy (6 mo before the visit).

A 61-year-old male patient visited the emergency room due to abdominal pain and concurrent hematemesis that started 2 days prior. Upon arrival, the patient’s vital signs were as follows: blood pressure, 112/63 mm Hg; heart rate, 106 beats per minute; respiratory rate, 20 cycles per minute; body temperature, 35.0 °C; and oxygen saturation level with nasal prongs at 5 L/min, 97%. The patient reported a history of frequent vomiting attributed to chronic alcoholism. Further, he had been hospitalized 6 months back due to gastric ulcer-related bleeding and had received endoscopic interventions (clipping hemostat). With multiple gastric ulcerations, a gastric diverticulum was observed at the fundus on esophagogastroduodenoscopy at that time (Fig. 1). He also had a history of exploratory laparotomy and splenectomy for injuries sustained from a traffic accident that occurred over 30 years ago.

**Figure 2.**
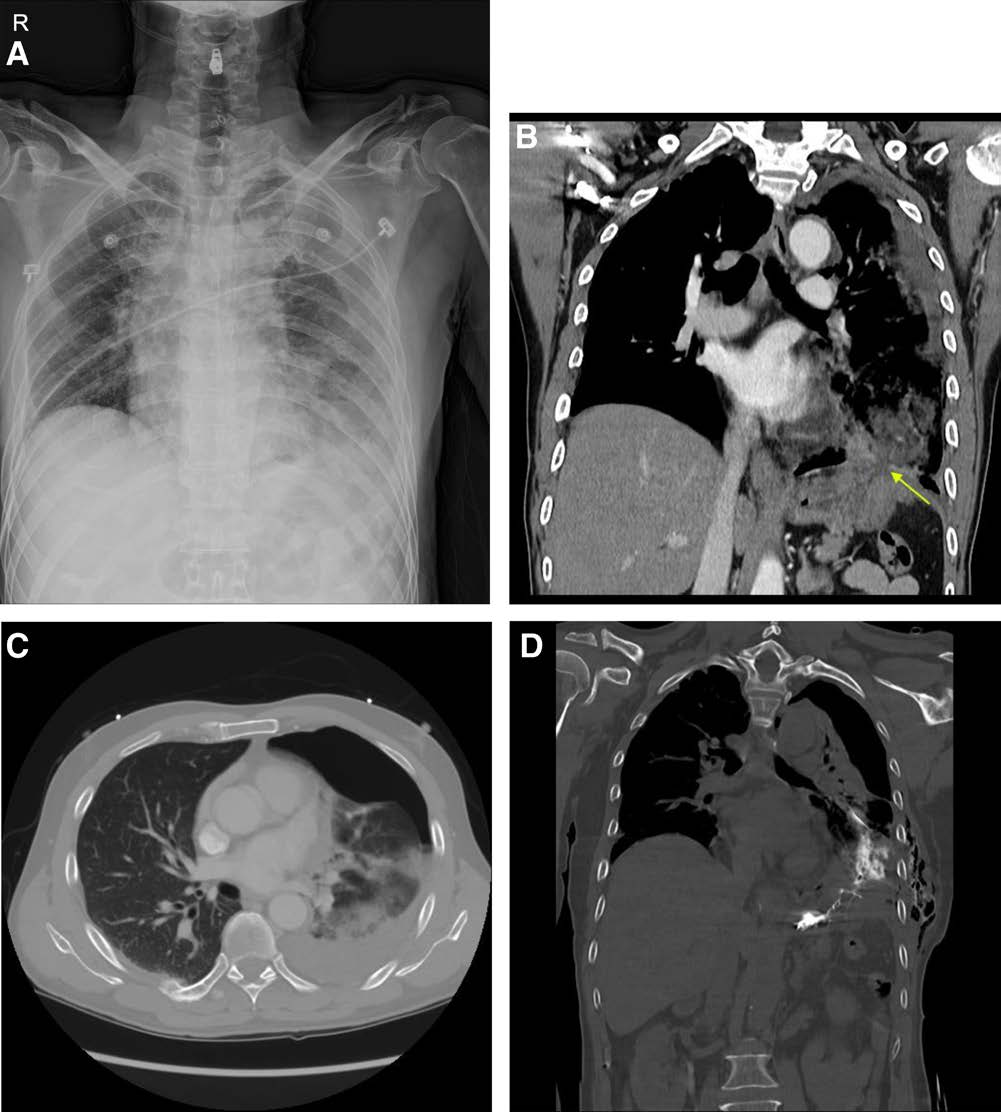
(A) Initial chest radiography. (B) Coronal view of contrast-enhanced chest computed tomography scan. Gastric, diaphragmatic rupture and communication with pleural space were suspected (yellow arrow). (C) Axial view of contrast-enhanced chest computed tomography scan. Left hydropneumothorax with pneumonia. (D) Noncontrast-enhanced chest computed tomography scan after oral contrast swallowing. Oral contrast was observed in the stomach and lung parenchyma.

Chest and abdominal computed tomography (CT) scan was performed at the emergency room. Results revealed left pneumothorax accompanied by fluid collection. Further, there was evidence suggesting the upward rupture of the stomach’s fundus above the diaphragm (Fig. 2B and C). A chest tube was inserted. Along with air leakage, a turbid fluid was drained. The fluid exhibited the following characteristics: white blood cell count, 193,000/µL; red blood cell count, 30,000/µL, with 80% polymorphonuclear cells and 20% lymphocytes; amylase level, 11,593U/L; and lactate dehydrogenase level, 1493 U/L. Exploratory surgery was planned immediately as gastric fistula into the thoracic cavity was suspected. To identify the point of leakage, the patient swallowed an oral contrast agent, and noncontrast-enhanced chest CT scan was performed. That is, 50 mL of iopromide, and water was mixed. Half of the mixture was swallowed 30 mins before imaging, and the other half just before imaging. Paraesophageal and intrapleural contrast leakage was not observed. The contrast was detected within the stomach and lung parenchyma, leading to the diagnosis of GPF (Fig. 2D).

**Figure 3.**
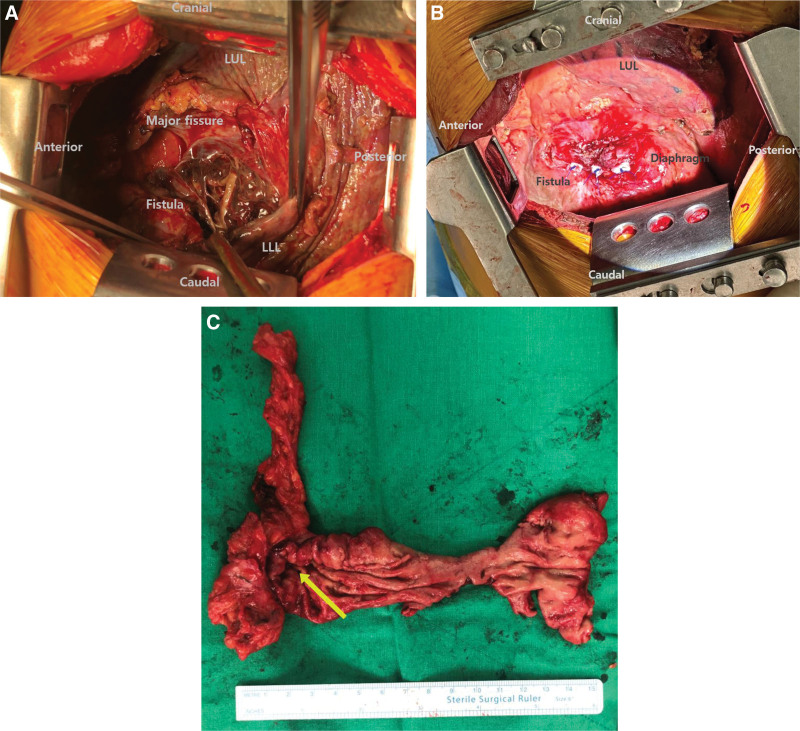
(A) The fistula opening was observed on the major fissure via the LLB. (B) The diaphragmatic opening was closed using pledgeted 2-0 prolene sutures in 3 interrupted stitches for primary closure. (C) The resected portion of the stomach. A transdiaphragmatic opening was observed (yellow arrow). LLB = left lower lobe.

Under general anesthesia, using a double-lumen endotracheal tube, the patient was positioned in the right lateral decubitus position. Left posterolateral thoracotomy was performed via the 6th intercostal space with the division of the 7th rib. Pus discharge and infected necrotic tissue were removed. Then, extensive irrigation was conducted. Moderate pleural adhesions were present, and they were released via electrocautery. When necrotic tissue was removed from the major fissure, the fistula was observed on the fissure via the LLL (Fig. 3A). For necrotized and infected LLL, lobectomy was performed. There was a diaphragmatic opening with a diameter of 2.5 cm (Fig. 3A), which was closed using pledgeted 2-0 prolene sutures in 3 interrupted stitches for primary closure (Fig. 3B). Next, 2 chest tubes (28-Fr straight and a curved one) were inserted. Then, the patient’s position was changed to supine, and an abdominal midline incision was made. Adhesiolysis, followed by partial gastrectomy, was performed (Fig. 3C). After the surgery, there were no signs of bleeding, and the patient’s vital signs remained stable. Postoperatively, piperacillin, and tazobactam were administered for complex intraabdominal infection. Initial culture samples collected from the chest tube drainage and intraoperative purulent discharge did not yield identifiable bacterial pathogens.

On postoperative day 1 (POD 1), the patient underwent extubation using the ventilator weaning protocol. On POD 2, chest radiography revealed haziness in the right lung. Due to the patient’s inability to effectively cough and atelectasis progression, oxygen supplementation with a high-flow nasal cannula was administered. However, the patient then developed fever, and chest radiography performed on POD 3 showed pneumonia. Hence, the antibiotics were changed from piperacillin and tazobactam to combined meropenem and levofloxacin. On POD 5, due to worsening pneumonia, the patient underwent reintubation. After modifying antibiotic treatment and administering intensive ventilator care, the patient’s clinical condition improved. Starting on POD 8, continuous feeding with water at a rate of 20 mL/h was initiated via a nasogastric (NG) tube. After feeding, there were no abnormalities observed in the chest tube drainage and laparotomy site pen rose drainage. Consequently, the water feeding rate was gradually increased to 40 mL/h. On POD 12, enteral feeding formula (Harmonilan^®^) was started at a rate of 20 mL/h. Despite improvement in pneumonia with ventilator care and antibiotic treatment, the patient could not be successfully weaned from the ventilator. Therefore, tracheostomy was performed on POD 14. On POD 20, there was improvement in pneumonia on chest radiography. However, the patient presented with abdominal wound dehiscence with pus-like discharge and fever. Laboratory tests revealed an elevated C-reactive protein (CRP) level at 111.9 (reference range: < 5.0) mg/L. To further assess the cause of inflammation, contrast-enhanced abdominal CT scan was performed. Although a prominent focus of infection was not identified on CT scan, the daily drainage from the laparotomy site’s (>400 mL; Penrose drain) and signs of infection at the laparotomy site were observed. Hence, possible intraabdominal wound infection was suspected, and a collaborative approach with the infectious disease department was used. With fasting, the antibiotics were changed from meropenem and levofloxacin to tigecycline and amikacin. In addition, a vacuum-assisted closure system was applied to the infected skin wound. The patient’s CRP levels decreased to 33.2 (reference range: < 5.0) mg/L, and positive clinical improvements were observed. Thus, on POD 26, ventilator weaning, and oxygen treatment with a high-flow nasal cannula were initiated. With decreased drainage volume and improved wound dehiscence, NG tube feeding was resumed. After the oral water intake test, the NG tube was removed on POD 33, and soft diet was initiated. After the infection was successfully controlled and the patient had favorable clinical progress, the intravenous antibiotics were discontinued and transitioned to oral amoxicillin and clavulanate. On POD 56, the tracheostomy tube was removed, and the patient was discharged.

This study was approved by the Institutional Review Board (IRB approval no., CR-23-120), and the need for informed consent was waived.

## 3. Discussion

Janilionis et al reported a case study of GPF that occurred due to invasive fungal infection after chemotherapy for multiple myeloma.^[3]^ In the current case, although the actual underlying mechanism remains uncertain, the patient had a previous history of exploratory laparotomy and splenectomy for injuries sustained from a traffic accident that occurred a long time ago. In addition, the patient visited the hospital 6 months prior due to hematemesis. At that time, a diverticulum in the fundus was suspected on esophagogastroduodenoscopy examination (Fig. 1). Abdominal CT scan did not reveal any prominent evidence of gastric fistula or herniation. A gastric diverticulum can cause ulceration and perforation due to the retention of residual digestive juices within the mucosal sac.^[4,5]^ Chronic alcohol abuse can lead to gastritis and further progression of ulceration, potentially resulting in the development of a fistula.

If left untreated, GPF can be associated with a high risk of morbidity.^[6]^ Although conservative treatment with nothing by mouth, parenteral nutrition, antibiotics have been applied, they are usually unsuccessful. In most cases, resolving the communication via thoracotomy or laparotomy and excising the infected site may be beneficial.^[7,8]^ The surgical treatment options can vary based on the location and underlying cause of the fistula. The preferred approach is the thoracoabdominal approach, often involving partial left diaphragmectomy. Moreover, LLL lobectomy is commonly performed along with this procedure. The type of procedure (primary repair, local gastrectomy, total gastrectomy, or Roux-en-Y reconstruction) is selected based on the specific location and severity of the stomach fistula.^[6]^ Inserting a feeding jejunostomy tube can be helpful for providing adequate nutrition. In cases where there is a history of vomiting and concurrent pneumothorax, if there is no distinct gastropulmonary fistula or communicating tunnel evident on CT scan, a differential diagnosis between Boerhaave syndrome and simple gastropleural fistula can be challenging to obtain. In such a case, CT scan after oral contrast swallowing can be helpful in identifying the leakage point and obtaining an accurate diagnosis.^[6]^

Gastropulmonary fistula is a rare condition, and there is no consensus regarding its treatment. Although conservative treatment for GPF has successful outcomes based on several reports, Guillaud et al showed that 69% of patients with gastrobronchial fistula who received primary endoscopic treatment eventually required surgical intervention.^[9]^ Therefore, due to treatment failures, surgical intervention should be considered as the primary treatment option for GPF.

## Author contributions

**Conceptualization:** Tae-Hong Yoon, Yun-Ho Jeon.

**Data curation:** Chul Ho Lee, Yun-Ho Jeon.

**Investigation:** Han Sol Lee, Yun-Ho Jeon.

**Methodology:** Tae-Hong Yoon, Chul Ho Lee.

**Project administration:** Yun-Ho Jeon.

**Resources:** Chul Ho Lee.

**Software:** Tae-Hong Yoon.

**Supervision:** Tae-Hong Yoon, Yun Ho Jeon.

**Validation:** Chul Ho Lee.

**Visualization:** Tae-Hong Yoon.

**Writing – original draft:** Tae-Hong Yoon, Chul Ho Lee, Yun Ho Jeon.

**Writing – review & editing:** Tae-Hong Yoon, Han Sol Lee, Chul Ho Lee, Yun-Ho Jeon.
